# Effect of Tetracaine on Intraocular Pressure in Normal and Hypertensive Rabbit Eyes

**Published:** 2012-01

**Authors:** Ali Asghar Sarchahi, Hooman Bozorgi

**Affiliations:** Department of Clinical Studies, School of Veterinary Medicine, Shiraz University, Shiraz, Iran

**Keywords:** Glaucoma, Hypertensive Eye, Intraocular Pressure, Rabbit, Tetracaine

## Abstract

**Purpose:**

To evaluate the effect of tetracaine on intraocular pressure (IOP) in normal and hypertensive rabbit eyes.

**Methods:**

The study was conducted on 12 healthy rabbits as controls and 6 healthy rabbits in which an experimental model of ocular hypertension (OHT) was induced by administration of 70 mL/kg of tap water through an orogastric tube. One drop of tetracaine was instilled in the left eye while a drop of normal saline (placebo) was applied to the right eye of the control group. IOP was measured before and 0, 5, 10, 15, 20, 25, 30, 35 and 40 minutes after drop administration in this group. The OHT group also received one drop of tetracaine and normal saline in the left eyes and right eyes respectively, immediately after water loading; the instillation of drops was repeated after 55 minutes. IOP was measured before and 0, 5, 10, 15, 20, 25, 30, 35, 40, 55, 70, 85, 100 and 115 minutes after water loading in this group.

**Results:**

Tetracaine treated eyes in both groups (ocular hypertensive and normal controls) demonstrated significant IOP reduction at time zero (immediately after drop instillation) which was sustained up to 20 minutes, as compared to placebo treated eyes (P<0.05). In ocular hypertensive rabbits, repeat instillation of tetracaine significantly reduced IOP at 55 minutes up to 30 minutes thereafter.

**Conclusion:**

Topical tetracaine can reduce IOP; this fact should be considered in experiments evaluating IOP reducing agents.

## INTRODUCTION

Topical anesthetics are used for many diagnostic and therapeutic ocular procedures such as intraocular pressure (IOP) measurement in humans and animals.^1^–^4^ The two most commonly used topical anesthetics are proparacaine and tetracaine.^4^ It has previously been reported that topical anesthetics may reduce IOP.^1^,^2^,^5^–^7^ Although these agents are commonly used in everyday practice, the use of topical anesthetics may interfere with results of investigations designed to evaluate the effect of different drugs or interventions on IOP.

Experimental ocular hypertension (OHT) can be created by administration of tap water via an orogastric tube, which induces acute ocular hypertension lasting up to 2 hours. This model is used for evaluating drugs that act on outflow facility or the production of aqueous humor.^8^ To the best of our knowledge, there are no reports on the effect of tetracaine on IOP. This study was designed to evaluate the effect of tetracaine on IOP in healthy rabbit eyes and to compare it to the corresponding effect in an experimental model of ocular hypertension.

## METHODS

Twelve adult white New Zealand rabbits, including six male and six female animals, weighing 1–3 kg (mean weight, 1.77±0.53 kg) with normal eye examinations including slit lamp biomicroscopy, ophthalmoscopy and tonometry were used for the purpose of this study. The study was approved by the Research Animal Care and Use Committee at the School of Veterinary Medicine, Shiraz University and complied with the ARVO Statement for the Use of Animals in Ophthalmic and Vision Research. The animals were maintained for one week in a standard room on a 12 hour light-dark schedule to adjust to the environment and were provided with food and water ad libitum. During this period, IOP was measured daily to make the animals familiar with tonometry.

In order to test the drug in healthy rabbits, one drop of 0.5% tetracaine hydrochloride (Anestocaine, Sinadaru, Iran) was instilled in the left eyes (normal group, treatment eye) and one drop of normal saline was applied in the right eyes (normal group, placebo eye). IOP was measured before and 0, 5, 10, 15, 20, 25, 30, 35 and 40 minutes after drop administration using an electronic applanation tonometer (Tonopen VET, Reichert Inc., NY, USA). In order to study the effect of tetracaine in conditions of abnormality, ocular hypertension was induced in six healthy rabbits by the administration of 70 mL/kg of tap water via an orogastric tube. The left eyes of this group received one drop of tetracaine 0 and 55 minutes after water loading (OHT group, treatment eye) and the right eyes received a drop of normal saline at the same intervals (OHT group, placebo eye). IOP was measured before and 0, 5, 10, 15, 20, 25, 30, 35, 40, 55, 70, 85, 100 and 115 minutes after water loading. The time at which the palpebral reflex was observed (return of ocular sensation), was also recorded.

Paired *t*-test was used to compare mean IOP between the study groups. P values less than 0.05 were considered as statistically significant.

## RESULTS

### Diurnal variation

**Figure 1.**
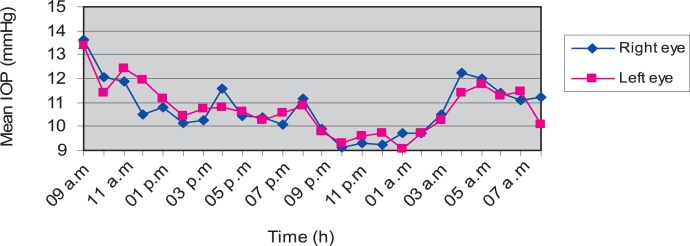
Diurnal variation of mean intraocular pressure (IOP) in normotensive rabbits. Maximum IOP occurred at 9 am and minimum IOP occurred at 10 pm. No significant difference was observed between the two eyes at any time point.

Mean diurnal IOP changes in healthy rabbits are displayed in figure 1. Maximum and minimum IOP values were recorded at 9 am and 10 pm, respectively in both eyes. Mean maximum IOP was 13.6±2.9 mmHg in right eyes and 13.4±3.0 mmHg in left eyes. Mean minimum IOP was 9.1±1.6 mmHg in right eyes and 9.3±1.7 mmHg in left eyes. No significant difference was observed between the two eyes of this group at any time point (P>0.05).

### IOP lowering effect of 0.5% tetracaine in normotensive eyes

**Figure 2.**
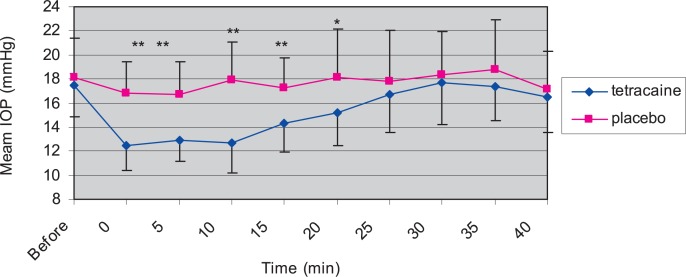
Effect of tetracaine on mean intraocular pressure (IOP) in 12 normotensive rabbits. Data are expressed in mean±standard deviations. Tetracaine was instilled in treated eyes at time zero. IOP decreased immediately after tetracaine instillation lasting for 20 minutes in treated eyes (*P<0.01, **P<0.001).

Mean baseline IOP values before drop instillation in treated and placebo eyes of the normal control group were 17.5±2.6 and 18.1±3.3 mmHg, respectively (P>0.05). At time zero (immediately after drop instillation), mean IOP of treated eyes reached 12.5±2.1 mmHg reflecting a 28.6% decrease from baseline values (P<0.001). This IOP reduction continued up to 10 minutes after drug administration and then started to increase, but was significantly lower than baseline up to 20 minutes after drug administration (P<0.01). When compared to placebo eyes, IOP reduction in treated eyes was significant at time zero until 20 minutes after drug instillation (P<0.01). The peak IOP reducing effect of tetracaine (5.2 mmHg, 29.1%) was observed 10 minutes after instillation (Fig. 2). Mean IOP reduction in treated eyes as compared to placebo eyes at times 0, 5, 10, 15 and 20 minutes was 4.3 (25.6%), 3.8 (23.2%), 5.2 (29.1 %), 3 (17.3%), and 3 (16.8%) mmHg, respectively.

### IOP lowering effect of 0.5% tetracaine in ocular hypertensive eyes

**Figure 3.**
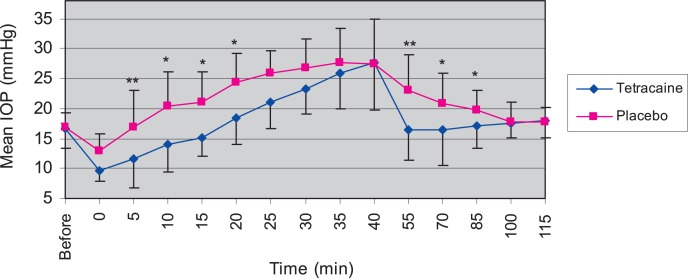
Effect of tetracaine on intraocular pressure (IOP) in 6 ocular hypertensive rabbits. Data are expressed in mean±standard deviations. Tetracaine was instilled in treated eyes 0 and 55 minutes after water loading; IOP decreased immediately after tetracaine instillation up to 20 and 30 minutes later, respectively (*P<0.05, **P<0.01).

As shown in figure 3, mean baseline IOP values of treated and placebo eyes before water loading were 16.7±3.3 and 16.8±2.5 mmHg (P>0.05), respectively. At time zero (immediately after water loading and drug instillation), mean IOP decreased to 9.7±1.8 mmHg (41.3% reduction, p=0.001) in the treated group and to 12.8±3.0 mmHg (23.8% reduction, P=0.012) in the placebo group. Thereafter, mean IOP started to increase in both eyes, but the rate of IOP increase in treated eyes was lower than that in placebo eyes. Mean IOP in placebo eyes increased to 24.3±4.8 mmHg (44.6%) 20 minutes after water loading (P=0.009) and reached its maximum (27.7± 5.7 mmHg, 64.9% increase) 35 minutes after water loading. It then decreased gradually and reached baseline values 70 minutes later. Mean IOP in treated eyes, similar to placebo eyes, increased gradually but at a significantly lower rate 5, 10, 15, and 20 minutes after water loading. Mean IOP rise in treated eyes at the above-mentioned intervals was 5.1 (30.4%), 6.3 (31%), 5.8 (27.6%) and 5.8 (23.9%) mmHg, respectively. Another drop of tetracaine was instilled in the left eyes 55 minutes after water loading to investigate the effect of tetracaine on pre-increased IOP. At this time, a sudden drop of IOP to 16.3±5 mmHg was observed (6.7 mmHg reduction, 29.1%, P=0.026,) in treated eyes as compared to placebo eyes. IOP remained significantly lower in treated eyes as compared to placebo eyes up to 85 minutes (P<0.05).

Loss of corneal sensation (eyelid blinking) was observed immediately after instillation of tetracaine in both normotensive and hypertensive eyes. Corneal sensation returned 20 and 22 minutes after drug administration in normotensive and hypertensive eyes, respectively (P>0.05).

## DISCUSSION

Tetracaine is a potent ester-type local anesthetic agent. It is usually used topically in ophthalmology; it has also been used for spinal anesthesia.^4^,^9^ It acts by reversibly stabilizing voltage-dependent sodium ion channels, decreasing their permeability to sodium ions and thus blocking the initiation and conduction of sensory nerve impulses to the spinal cord and brain stem.^2^,^4^

In the present study, topical tetracaine led to IOP reduction in both normotensive and hypertensive eyes in an experimental rabbit model. IOP decrease in both groups was observed immediately after drug instillation and was continued up to 20 minutes later. The absolute level of IOP reduction in hypertensive eyes exceeded that in normotensive eyes, but no difference was noted in the percentage of reduction. This may be due to higher baseline IOP in hypertensive eyes.^8^ Repeat instillation of tetracaine at 55 minutes, resulted in a further significant IOP reduction which was continued up to 30 minutes thereafter. This observation shows that tetracaine, in addition to preventing IOP rise, can also reduce established high IOP.

To the best of our knowledge, there are no other reports on the effect of tetracaine on IOP in humans or animals and the exact mechanisms of its effect are unclear. Some reports have shown that other topical anesthetics can reduce IOP.^1^,^2^,^5^–^7^ Baudouin and Gastaud^1^ reported significant IOP reduction using oxybuprocaine and betoxycaine. They reported IOP reduction to start one minute after instillation and to be continued for at least 15 minutes which is similar to our results.

Some studies have reported a relationship between IOP and central corneal thickness.^10^,^11^ Asensio et al^5^ reported that the IOP-lowering effect of oxybuprocaine is due to a decrease in corneal thickness, whereas in the study by Montero et al,^6^ IOP decreased without any change in corneal thickness. The latter investigators used a non-contact tonometer and reported that a combination of tetracaine and oxybuprocaine caused a significant reduction in IOP up to 5 minutes after drug instillation. They also measured corneal thickness and reported that this combination of topical anesthetics did not alter corneal thickness.

In contrast to these reports, in a study by Ehongo et al^3^ who used oxybuprocaine and measured IOP using a contact tonometer, IOP did not significantly change as compared to baseline values two minutes after drug instillation.

Some reasons that may explain the mechanism of IOP reduction by topical anesthetics include reduced lid squeezing^6^,^12^ or breath holding by the patient in preparation for the procedure,^6^ softening of the cornea by topical anesthesia,^13^ and relaxation of the eyeball and ciliary muscles (since extraocular muscle tone and scleral rigidity can also affect IOP)^14^.

The actual effect of topical anesthetics on IOP can only be demonstrated by non-contact tonometers that do not require the use of anesthetics.^6^ Though non-contact tonometers have gained widespread acceptance, the consensus is that contact tonometry is more accurate and should be employed to confirm readings obtained by the former methods and is the preferred technique for IOP measurment.^2^ In the present study, we used a contact tonometer (Tonopen VET, Reichert Inc., NY, USA) which is an easy-to-use, hand-held instrument that provides accurate IOP readings for veterinary uses.^15^,^16^ As topical anesthetics were not used in some eyes (placebo eyes), there were some cases of eyelid blinking, but the contact tonometer was used with no problem and did not cause major discomfort for the rabbits; furthermore, no considerable changes were observed in IOP throughout the study.

In summary, it seems that topical tetracaine seems to reduce IOP and therefore may interfere with the results of experiments that evaluate the effect of medications or interventions on IOP. In these studies, part of the IOP reduction may be due to the topical anesthetic, not the investigated agent.
